# Sex-related refractive disparities in school-aged children: a population-based cross-sectional study of 4.47 million screening records

**DOI:** 10.3389/fpubh.2026.1983884

**Published:** 2026-10-20

**Authors:** Dong-kan Li, Chun-hong Chen, Xiao-fang Zhan, Bin Lin, Xiao-feng Li

**Affiliations:** 1 Xiamen Eye Center and Eye Institute of Xiamen University, School of Medicine, Xiamen, China; 2 Xiamen Clinical Research Center for Eye Diseases, Xiamen, Fujian, China; 3 Xiamen Key Laboratory of Ophthalmology, Xiamen, Fujian, China; 4 Fujian Key Laboratory of Corneal & Ocular Surface Diseases, Xiamen, Fujian, China; 5 Xiamen Key Laboratory of Corneal & Ocular Surface Diseases, Xiamen, Fujian, China; 6 Translational Medicine Institute of Xiamen Eye Center of Xiamen University, Xiamen, Fujian, China

**Keywords:** generalized estimating equations, myopia, refractive development, school-based screening, sex differences

## Abstract

**Purpose:**

To delineate cross-sectional group-level sex-specific patterns of refractive development in school-aged children and to determine whether sex-related myopic disparities widen across age strata, addressing limitations in current large-scale epidemiological evidence.

**Methods:**

This population-based cross-sectional study analyzed 4.47 million valid vision screening records from 1,623,070 unique students (ages 3–18 years) across Southern Fujian, China (2021–2024). Spherical equivalent (SE) and uncorrected visual acuity (UCVA) were measured via non-cycloplegic autorefraction. Generalized estimating equations (GEE) with an exchangeable correlation structure (ICC = 0.473) were employed to account for repeated measures. A “sex × age” interaction term was incorporated into multivariable models to evaluate whether sex-related disparities widen across age strata. Sensitivity analyses compared encounter-level versus student-level analytic units.

**Results:**

While refractive parameters were comparable between sexes in the preschool group (≤6 years; SE difference: +0.04 D favoring females), a significant female disadvantage emerged in primary school (7–12 years; SE difference: −0.12 D), peaking in junior high school (13–15 years; SE difference: −0.20 D), followed by partial attenuation in senior high-school (≥16 years; SE difference: −0.17 D) in two of three cities. By senior high school (≥16 years), the female myopia prevalence was 88.7% versus 80.4% in males (absolute difference: 8.3 percentage points), despite partial convergence in continuous SE values. Within our limited set of available covariates (adjusted for age and city), GEE models showed female sex was associated with an overall SE difference of *β* = −0.141 D (95% CI −0.154 to −0.128 D), higher visual-impairment risk (OR = 1.26, 95% CI 1.22–1.30), and higher myopia risk (OR = 1.18, 95% CI 1.11–1.25), with a significant “sex × age” interaction (*p* < 0.001). Sensitivity analyses confirmed robustness, with SE differences fluctuating <0.05 D across analytic units.

**Conclusion:**

This study reveals distinct sex-related disparities in refractive status across childhood, observed in cross-sectional group-level comparisons. These group-level patterns may reflect developmental influences and potential cohort-related effects from differing historical exposures. These findings underscore the necessity of incorporating sex as a stratification factor in public health strategies. Implementing earlier and more intensive screening protocols for girls is crucial for curbing myopia progression and achieving life-course visual health equity.

## 1 Introduction

Myopia in children and adolescents has emerged as a global public health priority, with projections indicating that nearly half of the world’s population will be affected by 2050 (1–3). Although the high prevalence of myopia among children in East Asia is well established, large-scale epidemiological studies have largely focused on estimating average prevalence or characterizing urban–rural gradients, often treating sex as a covariate rather than an effect modifier of the tempo of myopia onset and progression (4). This analytical convention may underestimate the long-term implications of refractive development during childhood for visual health in adulthood. Notably, despite the emphasis on myopia control in pediatric populations, an 8-year cohort study of 691 young adults demonstrated that refractive error continued to progress at a rate of 0.04 D per year and axial length continued to increase even during early adulthood (ages 20–28), with a cumulative myopia incidence of 14% over the follow-up period; stratified analyses further revealed that females exhibited a significantly higher rate of refractive progression than males (5). Given that sex differences in refractive status during early adulthood likely originate from developmental trajectories in school-aged children, delineating whether girls manifest a more aggressive refractive drift during development is critical for life-course myopia management.

The existence of sex-specific refractive developmental trajectories is biologically plausible to a certain extent. First, girls enter puberty earlier than boys, and pubertal somatic development exhibits distinct sexual dimorphism (6). Given that the most rapid phase of axial elongation occurs during adolescence, sex differences in somatic maturation are hypothesized to contribute to the observed sexual heterogeneity in refractive development. Second, estrogen receptor signaling modulates extracellular matrix remodeling in mesenchymal cells (7); based on this biological premise, this pathway is postulated to participate in scleral remodeling and potentially mediate the more rapid myopic shift observed in females. Third, converging evidence from behavioral epidemiology indicates that, within the context of intensive academic pressure in Asia (8), boys generally exhibit higher levels of physical activity (9–11). In contrast, girls tend to engage in more sedentary behaviors during school hours. As outdoor time is typically coupled with dynamic physical activity, these sex differences indirectly support a behavioral pattern in which girls may have less outdoor exposure and a potentially higher burden of near-work activities. However, these mediating factors are rarely measured concurrently with refractive outcomes in large-scale epidemiological studies.

Despite the widespread implementation of school-based vision screening programs worldwide, three critical gaps remain in the current evidence on sex disparities. First, data on sex and age interactions are lacking; most sex-stratified studies are derived from small clinical samples or single-center cohorts, precluding a fine-grained characterization of how sex differences evolve across the full 3–18-year age spectrum. Second, few investigations have examined changes in spherical equivalent (SE) within a sex-stratified framework. Third, in the context of ultra-large real-world datasets, the sensitivity of sex effects to the choice of analytic unit (encounter-level versus student-level) and clustering structures remains insufficiently validated.

To address these gaps, the present study used 4.47 million school-based vision screening records from Southern Fujian, China (2021–2024) to provide the first systematic delineation of cross-sectional, group-level, sex-specific patterns of refractive status across all age strata. Using generalized estimating equations (GEE) to account for repeated measures, we quantified age-stratum-specific differences in SE between sexes and, crucially, tested the “sex × age” interaction to evaluate whether sex-related disparities differ across successive age groups. Furthermore, by comparing analyses conducted at the encounter level versus the student level, we verified the robustness of sex effects within a large-scale screening ecosystem. These findings aim to inform the development of sex-stratified, life-course myopia prevention and control strategies supported by high-level epidemiological evidence.

## 2 Materials and methods

### 2.1 Study design and data source

This study conducted a cross-sectional analysis using large-scale, school-based vision screening data. The raw data were derived from routine vision screening programs conducted between 2021 and 2024 across Southern Fujian, China (Xiamen, Quanzhou, and Zhangzhou). A total of 4,475,132 valid screening records were included, covering 1,361 schools and involving 1,623,070 unique students.

A related but methodologically distinct manuscript from the same screening database focuses on regional disparities and correction gaps in refractive errors. The present study utilizes the same raw dataset. Still, it addresses a different scientific question—sex-specific refractive trajectories—and does not reuse any result tables or figures from the companion paper. Overlapping descriptive statistics are limited to baseline denominators.

A STROBE-compliant participant flow diagram detailing record screening, exclusion criteria, and missing-data patterns for this identical dataset has been published elsewhere [see Supplementary Figure S1 in Li et al. (12)].

### 2.2 Ethical statement

This study strictly adhered to the principles of the Declaration of Helsinki and was approved by the Ethics Committee of Xiamen Eye Center of Xiamen University (Approval No. XMYKZX-KY-2023-004). Given that the original screening constituted a routine public health service, the Ethics Committee granted a waiver of informed consent for this retrospective analysis of anonymized data.

### 2.3 Visual and refractive measurements

Uniformly trained technicians performed all screenings. Uncorrected visual acuity (UCVA) was assessed using a standard Chinese 5-point logarithmic visual-acuity chart. On this scale, 5.0 corresponds to decimal visual acuity 1.0 and a logMAR value of 0.0. In the present study, following routine Chinese school-vision-screening conventions, visual impairment was defined as UCVA < 5.0 (i.e., decimal acuity < 1.0). This screening-based threshold does not directly match WHO clinical visual-impairment classification criteria, which use stricter cut-offs for functional visual loss. Non-cycloplegic refractive examinations were conducted using a Topcon KR-8900 autorefractor (Topcon Corporation, Tokyo, Japan), which was calibrated annually according to the manufacturer’s specifications. The SE was calculated as: SE = sphere + 1/2 × cylinder. Myopia was defined as SE ≤ −0.50 diopters (D).

### 2.4 Variable definitions

The primary outcome variable was SE. The core independent variables were sex (male/female) and age group (preschool: ≤6 years; primary school: 7–12 years; junior high school: 13–15 years; senior high school: ≥16 years). City was included as a covariate to adjust for regional heterogeneity.

### 2.5 Statistical analysis

Given the clustered nature of the data (multiple records per student), the following analytical strategies were employed:

Descriptive Analysis: SE means and standard deviations, along with myopia prevalence, were calculated, stratified by sex and age group. Group comparisons utilized analysis of variance (ANOVA) for continuous variables and the chi-square test for categorical variables.

Multivariable Regression Models: To account for repeated measures, GEE with an exchangeable working correlation matrix were constructed, clustering by student ID to obtain cluster-robust standard errors. A linear GEE model assessed the effect of sex on SE, adjusting for age and city. A logistic GEE model assessed the effect of sex on myopia risk. A “sex × age” interaction term was incorporated to test whether the magnitude of sex-related refractive disparity varied across successive age strata. Significance was evaluated via the respective model tests.

Sensitivity Analysis: To validate robustness, the analytic unit was restricted from “encounter-level” to “student-level” (using the most recent valid measurement per student), and the models were refitted. Consistency in the direction and magnitude of effects between the two approaches confirmed the stability of the findings.

Missing-data handling: Spherical-equivalent analyses included all records within the final analytical dataset, as valid autorefraction measurements were available for every entry. Analyses of uncorrected visual acuity were restricted to records with non-missing right-eye visual-acuity values (4.9% missing). Missing uncorrected visual-acuity occurred predominantly among preschool-aged children due to poor examination cooperation. We did not perform multiple imputation; analyses used complete-case subsets for each outcome.

Software and Significance: All analyses were performed using SPSS Statistics version 22.0 (IBM Corp., Armonk, NY, USA). A two-tailed *p* < 0.05 was considered statistically significant. Given the large sample size, in addition to *p* values, effect sizes (e.g., mean differences, odds ratios [OR]) were reported to evaluate clinical significance.

## 3 Results

### 3.1 Distribution of refractive parameters by sex and age group

Among the 4,475,132 valid screening records, 53.9% were from male students, 45.7% from female students, and 0.4% lacked sex information. Table 1 reports uncorrected visual-acuity statistics restricted to the subset with available right-eye uncorrected visual-acuity measurements (*n* = 4,237,528; 4.9% missing for uncorrected visual-acuity). Descriptive analysis revealed that sex disparities in refractive parameters exhibited a dynamic evolution with age.

**Table 1:** Sex-specific differences in uncorrected visual acuity across age groups.

Age group	Male mean (*N*)	Female mean (*N*)	Difference value (female–male)
Preschool (≤6 y)	4.877 (134,476)	4.876 (111,623)	−0.001
Primary (7–12 y)	4.794 (1,184,481)	4.765 (984,182)	−0.029
Junior High (13–15 y)	4.591 (633,822)	4.534 (533,644)	−0.057
Senior High (≥16 y)	4.507 (342,089)	4.455 (313,211)	−0.053

In the preschool group (≤6 years), pooled across all three cities, the female–male SE difference was +0.04 D (95% CI + 0.02 to +0.06 D, *N* = 326,601), with comparable mean SE between boys and girls, and no material difference in myopia prevalence (boys: 14.2%; girls: 14.5%). Starting with the primary school group (7–12 years), a sex disparity emerged: pooled female–male SE difference was −0.12 D (95% CI − 0.14 to −0.10 D, *N* = 2,235,613); girls had lower mean SE and higher myopia prevalence (62.3% vs. 58.1% in boys). This disparity peaked in the junior high school group (13–15 years), with a pooled female–male SE difference of −0.20 D (95% CI −0.21 to −0.19 D, *N* = 1,227,142). In the senior high-school group (≥16 years), the pooled female–male SE difference was −0.17 D (95% CI −0.19 to −0.15 D, *N* = 685,776). By senior high school, the myopia prevalence in girls remained significantly higher than in boys (88.7% vs. 80.4%), representing an absolute difference of 8.3 percentage points. The trend in UCVA paralleled that of SE, with girls consistently showing slightly lower mean UCVA values across all age strata than boys; this difference became particularly pronounced after puberty (Table 1; Figure 1). Table 2 presents disaggregated female-minus-male SE differences by city for each age stratum.

**Figure 1:**
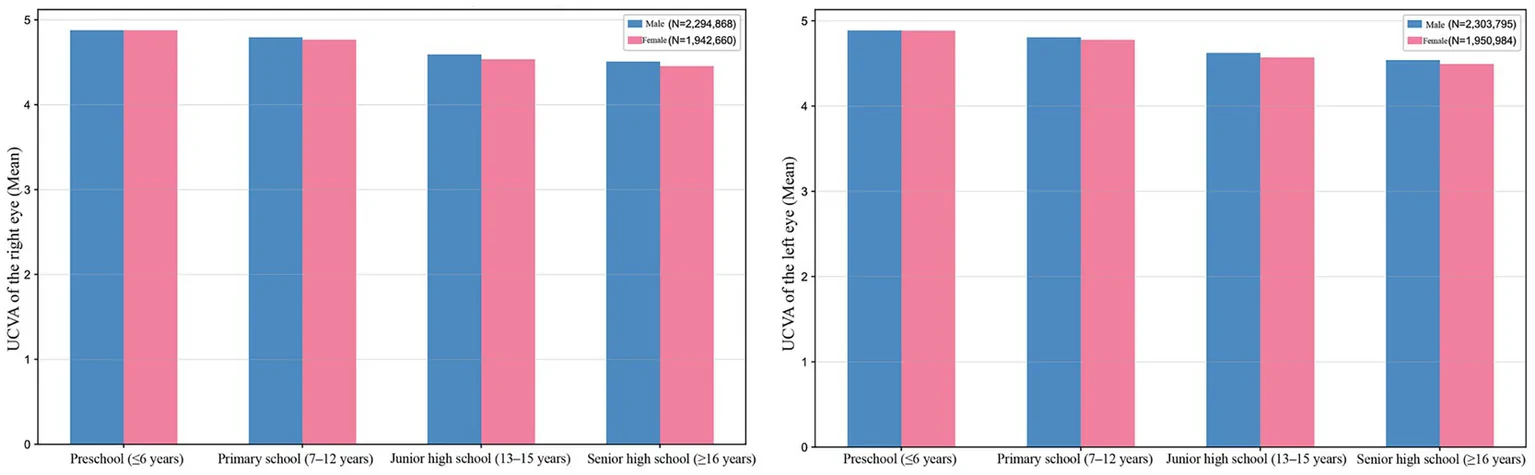
Uncorrected visual acuity stratified by sex and age group for right- and left-eye measurements. Data are restricted to participants with valid uncorrected visual-acuity measurements (*n* = 4,237,528). Bar charts display mean 5-point-scale uncorrected visual acuity (UCVA) for male (blue bars) and female (red bars) participants across four age strata: preschool (≤6 years), primary school (7–12 years), junior high school (13–15 years), and senior high school (≥16 years). The left subplot shows right-eye measurements; the right subplot shows left-eye measurements. UCVA was assessed using the standard Chinese 5-point logarithmic visual-acuity chart.

**Table 2:** Spherical equivalent differences under the interaction of sex and age.

Age group	Xiamen	Quanzhou	Zhangzhou
Preschool (≤6 y)	+0.02D	+0.05D	+0.06D
Primary (7–12 y)	−0.12D	−0.12D	−0.07D
Junior High (13–15 y)	−0.24D	−0.21D	−0.16D
Senior High (≥16 y)	−0.20D	−0.09D	−0.20D

Figure 1 illustrates the UCVA of the right and left eyes among male and female students stratified by age group.

Table 2 presents the sex-stratified SE differences (female SE minus male SE) under the interaction effect of sex and age across all cities. While females exhibited a slight refractive advantage over males during the preschool stage, this pattern reversed upon entry into primary school, with females demonstrating a consistent myopic disadvantage that peaked during junior high school. This disadvantage persisted into senior high school. This pattern illustrates age-dependent sex-related refractive disparities, with peak divergence observed during junior high school.

### 3.2 Multivariable regression analysis

To account for age, city, and repeated within-student measurements, we constructed GEE models with an exchangeable working correlation matrix (ICC = 0.473). First, we fitted GEE models without the sex-by-age interaction term to estimate the overall sex-related effect across all age groups, adjusting for age and city. In the linear GEE model for spherical equivalent, female sex was associated with an overall mean SE difference of *β* = −0.141 D (95% CI −0.154 to −0.128 D). In logistic GEE models, female sex was associated with elevated risk of visual impairment (UCVA < 5.0; OR = 1.26, 95% CI 1.22–1.30) and higher myopia prevalence (OR = 1.18, 95% CI 1.11–1.25). Next, we added the sex-by-age interaction term. The interaction was statistically significant (*p* < 0.001), indicating that the magnitude of sex-related refractive disparity differed across successive age-groups. All GEE analyses could not adjust for important unmeasured educational-behavioral covariates.

### 3.3 Sensitivity analysis

To validate the robustness of the findings, the unit of analysis was restricted from “encounter-level” to “student-level” (using the most recent valid measurement per student). In this sensitivity analysis, the direction of the sex disparity remained entirely consistent with the primary analysis. The intergroup difference in mean SE fluctuated by less than 0.05 D, and the difference in prevalence rates was less than 2 percentage points. These findings indicate that the core conclusions regarding sex disparities are not contingent upon the choice of analytic unit (encounter-level vs. student-level).

## 4 Discussion

Leveraging 4.47 million school-based vision screening records from Southern Fujian, China, this study systematically delineates sex-specific refractive developmental trajectories among children and adolescents aged 3–18 years. Our findings consistently show that while refractive status is largely comparable between sexes during the preschool years, females show a greater myopic shift (lower spherical equivalent, SE) and higher myopia prevalence starting in primary school. Sex-related SE disparity generally peaks in junior high school. It partially attenuates in senior high school across two of three cities, even though myopia-prevalence differences remain substantial at older ages. After adjusting for age and city in multivariable GEE models, sex remained statistically associated with refractive outcomes, and the “sex × age” interaction term was statistically significant. However, this association may be partly driven by unmeasured education-related exposures. These findings reveal a distinct vulnerability in female refractive development during adolescence, providing novel epidemiological evidence for understanding the sex disparity in the myopia epidemic.

### 4.1 Biological basis of sex-specific trajectories

The observed peak sex-related refractive disparity at 13–15 years may partly reflect a pubertal phase-shift (temporal displacement) between sexes, rather than a purely intrinsic, permanent female vulnerability, which aligns with the earlier onset of puberty in girls (13). As noted in the Introduction, estrogen receptor signaling modulates extracellular matrix remodeling (7), and pubertal somatic development exhibits marked sexual dimorphism (6). Girls enter pubertal somatic development approximately 1.5–2 years earlier than boys, with divergent tempo and duration of pubertal progression. Since the period of fastest ocular axial elongation coincides with pubertal growth spurts, comparison by chronological age juxtaposes girls already within the high-risk window for myopia development against boys who have not yet entered this developmental phase. The sex-by-age interaction detected in our GEE models is therefore compatible with this developmental-timing-driven phase-shift model: the peak disparity observed at 13–15 years could arise because girls reach the period of rapid axial elongation earlier, even if underlying biological susceptibility does not permanently differ between sexes. Estrogen-related scleral remodeling pathways described above may still contribute to refractive change during puberty. Still, without biological maturation markers, we cannot disentangle the effects of pubertal timing from inherent sex-specific biological susceptibility.

Partial attenuation of the SE-based sex gap in senior high school across most cities may reflect boys entering their own period of rapid ocular axial elongation and catching up with girls, consistent with a pubertal phase-shift framework.

It is important to emphasize that all refractive estimates in this work come from non-cycloplegic screening autorefraction. As documented in external multicenter data, non-cycloplegic measurements carry an age-dependent systematic bias that tends to overestimate population-level myopia prevalence, especially among younger children (14). This means the absolute myopia prevalence values reported herein should be interpreted cautiously. By contrast, our core inference centers on relative sex-comparisons across age and city strata. While sex-differential accommodative bias cannot be excluded, population-based evidence currently remains limited for systematic sex differences in resting accommodative tone. Replication with cycloplegic refraction would be required to confirm the magnitude of these sex-related refractive disparities definitively.

### 4.2 Behavioral modification of biological susceptibility

We note these behavioral pathways represent hypotheses derived from external literature and cannot be formally tested within our present dataset. Observed sex-related disparities could reflect a combination of intrinsic sex-related traits and gender-differentiated educational or lifestyle exposures. The substantial disparity observed in senior high school, where female myopia prevalence exceeded that of males by 8.3 percentage points, cannot be fully explained by biological factors alone. Converging evidence indicates that, within the context of intensive academic pressure in Asia, boys generally maintain higher levels of physical activity and moderate-to-vigorous physical activity (9–11), while outdoor light exposure is well-established as protective against myopia (15–18). As outdoor time is typically coupled with dynamic physical activity, whereas near-work is associated with sedentary behavior, the relatively higher sedentary time among girls during school hours likely translates to reduced outdoor light exposure and increased near-work burden (11). Although our screening dataset lacks individual-level behavioral metrics, these population-level patterns suggest that a more sedentary lifestyle may act as an effect modifier, amplifying the inherent biological susceptibility in females and thereby contributing to the elevated myopia risk observed.

### 4.3 Implications for public health strategy

Our findings indicate that females exhibit a lower SE and higher myopia prevalence starting from primary school, with this disadvantage becoming more pronounced across successive age strata. This suggests that, even under equivalent screening coverage, girls may face a disproportionately higher risk of uncorrected myopia and potential threats to visual development. Consequently, future public health strategies should incorporate sex as an independent dimension when determining screening frequency and intervention priorities, rather than relying solely on population-averaged visual acuity metrics.

### 4.4 Methodological robustness

This study used ultra-large real-world datasets and applied GEE models to account for within-student repeated-measure clustering, with an intra-class correlation coefficient of 0.473. This clustering statistic reflects the magnitude of correlation among multiple records originating from the same participant and motivated our choice of GEE, rather than serving as evidence for the validity of observed sex-related effects. Crucially, sensitivity analyses confirmed that the direction and magnitude of the sex effect on SE and myopia prevalence remained highly consistent regardless of whether analyses were conducted at the encounter-level or student-level (fluctuation in mean SE < 0.05 D; difference in prevalence < 2 percentage points). These findings refute the hypothesis that observed sex disparities arise merely from differential screening frequencies or data duplication. Instead, they demonstrate that the female refractive disadvantage is a stable phenomenon within large-scale screening ecosystems.

### 4.5 Limitations

Despite the substantial sample size, several limitations warrant consideration when interpreting our findings.

First, cycloplegic refraction data were not available. As a routine public-health screening tool, non-cycloplegic autorefraction suits large-scale surveillance but carries well-documented measurement bias: it tends to systematically overestimate myopia prevalence and underestimate hyperopia, with the largest distortion among younger children and hyperopic eyes. Published multicenter work using paired cycloplegic-non-cycloplegic data demonstrates that classification accuracy improves with age, whereas substantial misclassification risk persists in younger age groups (14). Although we focus primarily on relative sex-group differences rather than absolute population myopia prevalence, the absolute prevalence figures in this manuscript may overestimate the true cycloplegic-based myopia burden. The hypothesis that girls might exhibit greater accommodative tone shifting SE toward myopic values remains biologically plausible but lacks direct population-level empirical evidence; we cannot quantify how much, if any, of the observed sex disparity originates from sex-differential accommodative artefact. Thus, accommodative bias cannot be ruled out, but it is unlikely to fully explain the consistent, city-replicated, age-graded sex-related disparity pattern we observed.

Second, a cross-sectional design has inherent limitations. Our age-stratified comparisons reflect group-level differences across distinct birth cohorts rather than within-individual longitudinal progression over time. Although we identified a significant “sex × age” interaction, this snapshot design constrains causal developmental inferences, as age-related patterns cannot be fully disentangled from birth-cohort-specific exposures. Our screening window (2021–2024) overlapped with the COVID-19-associated surge in near-workload due to remote schooling, which may have impacted different birth cohorts unevenly and could contribute to observed age-stratum-specific sex disparities. As Enthoven et al. (4) highlight, the magnitude of sex-myopia associations can vary across birth cohorts; accordingly, our observed group-level disparity gradients may partly reflect generational shifts rather than purely age-dependent developmental processes. While the observed gradient across age strata suggests developmental trends, it does not provide definitive proof of within-child progressive change.

Third, individual-level behavioral and environmental data were lacking. We were unable to obtain specific information on outdoor time, near-work intensity, parental myopia history, or genetic background. Although we adjusted for age and city (as a proxy for socioeconomic status) and cited population-level behavioral evidence, we could not quantify the specific contribution of these mediators to the sex disparity at the individual level. Future prospective cohort studies should collect these covariates concurrently to disentangle the determinants of sex differences further.

Fourth, our analytical models lack direct covariates capturing educational intensity, near-work burden, or outdoor-time behavior. Sex may partly proxy these unmeasured behavioral-educational exposures, rather than exerting a fully independent causal effect. A published cycloplegic-refraction-based cohort study demonstrated that sex lost independent predictive power for myopia progression once educational-model variables were included in regression models (19). Our dataset does not contain school-level or district-level proxies for educational loading to further adjust for this potential confounding. Future studies with individual-level behavioral and educational data are needed to disentangle sex-related intrinsic factors from gender-linked environmental exposures.

Fifth, this screening dataset lacked biological markers of pubertal maturation, including menarche, Tanner staging, or peak height velocity. Without these proxies for biological age, we cannot align male and female participants by developmental stage. Consequently, our observed sex-by-chronological-age interaction cannot definitively disentangle effects driven by pubertal phase-shift (temporal displacement) from true inherent differences in refractive susceptibility between sexes, a limitation that would persist even if age were treated as a continuous variable in statistical models.

## 5 Conclusion

Based on 4.47 million screening records from Southern Fujian, this study reveals distinct sex-related disparities in refractive status across childhood, observed via cross-sectional group-level comparisons. Females showed a greater myopic shift, with lower spherical equivalent and higher myopia prevalence than males starting in primary school. Sex-related SE disparity peaked at junior high school. It partially attenuated in senior high school in two of three cities, while the myopia-prevalence gap remained considerable at 8.3 percentage points by senior high school. Within our available covariate set, adjusting for age and city, sex showed a statistical association with refractive status. Moreover, the significant sex-by-age interaction suggests a population-level window in which sex-related refractive disparity widens across successive age groups. These group-level patterns may reflect pubertal developmental-timing effects (phase-shift between sexes), other developmental influences and potential cohort-related effects from differing historical exposures. Although limited by non-cycloplegic refraction and a cross-sectional design, sensitivity analyses confirmed the robustness of these group-level disparities. These results underscore the necessity of incorporating sex into risk-stratification frameworks for future myopia control strategies, advocating for earlier and more intensive screening and intervention protocols targeting girls to promote life-course visual health equity.

## Data Availability

Template data collection forms, extracted data, analytic code, and other review materials are not publicly available due to institutional data management policies. Requests to access the datasets should be directed at: linbin1513@foxmail.com.
